# The genesis of perinatal clinical psychology and its contemporary issues

**DOI:** 10.3389/fpsyt.2023.1090365

**Published:** 2023-07-17

**Authors:** Sylvain Missonnier

**Affiliations:** ^1^Clinical Psychology, Institut de Psychologie, Université Paris Descartes, Boulogne-sur-Mer, France; ^2^Université Paris-Cité, Institut de Psychologie Laboratoire PCPP, Paris, Île-de-France, France

**Keywords:** clinical psychology, perinatality, parenthood, psychic reality, perinatal clinical psychology

## Abstract

The main aim of French clinical psychology is to explain the psychic processes of transformation, to which the subject is central. In this context, transformations in the perinatal period open an innovating field in perinatal clinical psychology focused on the conscious/subconscious, subjective/inter-subjective psychic reality of a subject who is in the process of becoming (or becoming once again) a parent and being born a human.

## 1. Introduction: clinical psychology

Clinical psychology is defined by its two-sided object:

- An exploration of the conscious/subconscious, subjective/inter-subjective psychic reality of the subjects’ day-to-day life in situation, in its individual/group, and normal/pathological forms.
- The implementation of formats of inter-subjective encounters favourable to the containing observation of verbal/non-verbal, focal/free associative processuality, and the implementation of the subjectifying meaning of its phenomenology and its conflicting inhibiting and dissociative obstacles.

The clinical methodology for this psychic reality is inseparable from its implementation across all participants involved: it is the inter-subjective encounter and its reciprocal effects that enables the exploration of virtual associations and a meaningful update of their contents.

Of course, this definition of clinical psychology is a skeleton which, to take flesh, must feed on constant ethical reflection and a multitude of modes (diagnosis, results, orientation, individual and group psychotherapies, supervision, expertise, etc.), belonging to the main classic domains of intervention by clinical psychologists: medicine, psychiatry, education and training, employment, justice, institutions, etc. inseparable from their specific cultural context.

Nonetheless, this attempt at a definition aspires to three decisive qualities:

- Highlighting clearly cardinal epistemological ‘values’ of clinical psychology (1–12).
- Being intrinsically open to inter-disciplinarity, so that the different specialties of care can appropriate this definition of clinical psychology and enrich it with their singularities and critical experience. If clinical psychology is the corporate monopoly of clinical psychologists alone, it betrays the foundations of its mixed identity, its true ‘mixed blood’; indeed, the permanent effort towards transition in practises and the multiplication of theories contributes to its history.
- Offering an appropriate framework to engage in its full application in the inter-disciplinary field of perinatal clinical psychology.

## 2. Perinatal clinical psychology

As an echo to this definition of clinical psychology, the perinatal period can be seen as an open window on the conscious/subconscious, subjective/inter-subjective psychic reality of the subject in a situation of becoming a parent and being born a human.

The process of perinatal parenthood corresponds to a particular amplification of bio-psychic transformations and the inherent processes of associativity/inhibition-dissociation. This parental metamorphosis corresponds, in extreme situations, to a potentially two-faceted crisis: traumatic and/or cathartic.

For better or for worse, parenthood, in transitory manner, highlights the typical elements of the structure and content of the subject’s psychic reality. Bydlowski (13), one of the first French psychoanalysts to have focused on perinatal clinical practise after Deutsch, Bibring, and Benedek, speaks of the pregnant woman’s ‘psychic transparency’.

However this ‘psychic transparency’, an element clinically and technically central to our definition of perinatal clinical psychology, is no doubt only virtual. The redeployment of its meaning^1^ is only a potential that may or may not be confirmed. The greater associativity inherent in the perinatal experience will only provide subectifying opportunities insofar as the internal and external characteristics of the subject’s psychic apparatus, the conjugal, familial, and professional inter-subjective spaces, along with the perinatal medical, psychological and social follow-up (maternity, neonatology, paediatrics, perinatal networks, etc.) are or are not favourable to its elaborative containing function. The constraints of this situation will be compounded in case of medically ‘pathological’ pregnancies and births, and/or in case of parenthood disorders and early parent/foetus/baby disharmony.

The first facet of the object perinatal clinical psychology will thus be an exploration of subjects’ subjective/inter-subjective, conscious/subconscious daily psychic reality in a situation of becoming a parent (or becoming a parent once more), of being born human and becoming carers, in all forms—normal/pathological, as an individual, a couple or a group.

The second facet of the object of perinatal clinical psychology is attention towards the scope for parents and caregivers to draw benefit from private, institutional surroundings favouring the metabolisation of verbal and non-verbal associative processuality (somatic, in particular), inherent in the bio-psychic transformations of parenthood and its professional care.

This containing clinical observation of what exists among the different participants and the varied stimulations deriving from the metabolising potential of verbal/non-verbal associativity corresponds to the strategies of primary medical, psychological and social prevention in perinatal clinical psychology. It has meaning and actual existence only in the setting of collegiate collaboration.

Possessing essential common grounds with primary prevention, secondary prevention concerns the offer of modes of inter-subjective encounters targeting parents and caregivers harbouring dissociative-inhibiting obstacles, thus hampering the metabolisation of verbal/non-verbal associative processuality and the attribution of meaning to subjective aspects of its phenomenology.

The inclusion of the ‘normal’ and the ‘psychopathological’ in clinical psychology, and thereby primary and secondary prevention, is essential to its perinatal version. As a result, as we will see, the history of perinatal clinical psychology demonstrates, in caricatural mode, how dissatisfactory and distressing it is for parents and their foetuses/babies to see attention directed solely to ‘noisy’ cases, which will merely receive one-off psychiatric responses.

The extension of the perinatal psychiatry and perinatal clinical psychology to the wide variations in perinatal setting in what is ‘normal’, and the extension to interdisciplinary caring attitudes more broadly (primary prevention), has been a source of humanisation of births and recognition of masked, mute or visible pathologies (secondary prevention).

Indeed, the ‘ordinary’ signs of suffering in the perinatal world are liable to be ignored or trivialised, and to evolve underground: the recurrence, still underestimated in the perinatal world, of anxiety, mood and psychosomatic disorders, family relationship disharmony, infant psychosomatic disorders, etc. easily demonstrates this cultural blind spot.

Denouncing the mirage of the conformist medical ‘normality’ of parenthood (i.e., defensively idealised and a cause of violent exclusions), primary prevention in the perinatal world is justified by a great individual variability in the nature, the content and the chronology of the parental anticipatory maturation in attitudes towards the foetus/baby (14). In response to the singularities of this ‘spontaneous’ parental prevention, institutional prevention aims to be ‘made to measure’. It thus adopts a humanistic approach by offering an interdisciplinary ritual framework, promoting recognition, shared anticipation and explanation for the potential shockwaves of this crisis, oscillating between vulnerability and creativity.

### 2.1. The epistemological foundations of perinatal clinical psychology

Perinatal clinical psychology is thus centred on the many psychological and psycho-pathological avatars of the encounter of ‘being born a human’, of ‘becoming a parent’ and of ‘being a caregiver’.

It unites perinatal psychology and perinatal psycho-pathology. The first studies the diversity of the tempered (but broad) formalisations of the process of attunement between the genesis of a subject and the accompanying process of parenthood. Perinatal psycho-pathology distinguishes the pathological patterns that perinatal psychology— in the strict sense—sets out to address in terms of prevention and care. Perinatal ethno-psychiatry (15) ponders about the cultural valency of the disharmony at hand, and its place in an appropriate therapeutic response.

Current perinatal clinical psychology was recently born out of interdisciplinary clinical practise, where psychiatry in the post-partum period plays an essential role. Its history is the best guide to understand the current issues in perinatal clinical psychology.

#### 2.1.1. Genesis

With (16), followed by his pupil (17), French psychiatry was initially innovative in the affirmation of the psychic specificity of the post-partum period (18). But it is only recently that perinatal psychiatry, essentially in Britain in the 1950s, appeared as an institutional practise (19). After a few pioneering experiments, the first mother-baby hospitalisation unit was opened in 1959 at Banstead Hospital in the United Kingdom, on the initiative of adult psychiatrists. This institutional formalisation was motivated by an immediate desire to not separate mothers from their newly born babies. In a population of psychotic mothers, Baker and his team, as early as 1961, reported encouraging retrospective results on the outcome of non-separated dyads from birth, compared to separated dyads. Since then, the dynamics of British perinatal psychiatry have never been refuted. To be convinced of this fact, one only has to mention the activities of the International Marcé Society for perinatal mental health, the first lectureship in perinatal psychiatry in London created by Kumar, or the work by Cox and Holden (20), a Scottish author, famous for a scale designed for the detection of maternal post-partum depression (the EPDS manual), which has been translated into French (21).

In France, it was Racamier who first organised mother-infant hospitalisations at Prémontré psychiatric hospital in northern France. Its concept of motherhood generated reflection in France on the psychic process of ‘becoming a mother’. However, it was not until 1979 that the first mother-baby hospitalisation unit was opened at Créteil inter-communal hospital. There are currently 20 full-time hospitalisation units and 11 day-units.^2^ The first national meeting of the different psychiatric teams offering joint hospitalisations was held in Créteil on January 29 and 30th 1993.

The contributions by Sutter and Bourgeois (19) and Dugnat (22, 23) provide a good overview of the environment of these units. They were, to start with, defined to host dyads, in which mothers either presented an overt, acute pathology of the post-partum period with its manic/depressive bipolarity (10–15% of parturient women), or an earlier psychiatric pathology or serious personality disorders. Young women with no psychiatric history were also hospitalised with their babies, because of difficulties building their identity as mothers and establishing an attachment favourable to their infant’s development.

Perinatal psychiatry was forged around the clinical care of psychotic, depressive and anxiety disorders and suicide risk among pregnant women and during the post-partum period. Today, whilst remaining faithful to this original line of approach, it is in particular extending its spectrum to a wider prevention perspective. Perinatal psychiatry can no longer merely apprehend overt, ‘noisy’ psycho-pathological disorders. It is opening up to the diversity of parenting dysfunctions—from the most masked to the most obvious, from the mildest to the most severe. This extension is particularly the result of incessant interaction between child and adult psychiatrists and psychologists involved in interdisciplinary clinical perinatal activities.

Perinatal clinical psychopathology is a co-construction between somatic and psychic specialists. For the latter, experience shared on a daily basis between psychiatrists and clinical psychologists who are involved in implementing primary and secondary prevention strategies in perinatal units, plays an essential role. On this point, it is interesting to observe how perinatal clinical practise is an area of great mutual creativity between psychiatrists (child and adult) and psychologists, in stark contrast with other sectors of mental health!

#### 2.1.2. The theoretical foundations of perinatal clinical psychology

In France, the psychology and psychopathology of very young children have been central in the claim for a specific identity of this new domain. On the interface between psychoanalytical and experimental observation, clinical practise in early infancy is progressively opening up to the complexity of the emergence of the psyche and inter-subjectivity. The light cast by theory of attachment of Bowlby (24–26) and by his successors, the reconsideration of the notion of self/other non-differentiation by new-born babies (27) and the essential realisation of the interactive dimension in babies’ postnatal bio-psychic development, have all led to innovating questioning on perinatal epigenetics.

In this line of thought, current perinatal psychopathology in France, combines, within an original referential space, contributions from ‘developmental psychoanalysis’ among infants (28) and research on the process of parenthood and its disorders.

- Developmental psychoanalysis is situated at the crossroads between studies on genesis and dysfunctions in relation to:

- the skills of the foetus/baby (29) and their interactive epigenetics (30);
- object relationships and attachment (31, 32);
- the emergence of the self and inter-subjectivity (Stern, 1989) (33–35);
- fantasised parent-baby interactions (36, 37) and inter/trans-generational transmission (38).
- triadification (39); and
- the psychosomatic functioning of the infant (40).

- Following on from theoretical founding research by English-language authors Deutsh (41), Benedek (42), Bibring (43), and Winnicott (44), the psychoanalytical approach to parenthood has a central role (14).

#### 2.1.3. The influence of a few pioneers

French perinatal clinical psychology is greatly indebted to certain clinicians, who were at first isolated, and whose innovating actions opened new paths that have since become emblematic. As a result, the current identity of this practise is to be situated in the wake of the first psychoanalysts first involved in paediatrics and then in maternity and neonatology.

Raimbault inaugurated this approach in the 60s. Royer, who managed the paediatric nephrology unit in the Hôpital des Enfants Malades (Paris), asked him to join his team. This collaboration, which was radically original in those days, led to the creation of the first research unit to include psychoanalysts and sociologists. The ‘*Clinique du réel*’ (45) (real-life clinical practise) was born from this encounter between psychoanalysis and medical paediatrics.

It is clear that the theme of child death here forms an institutional diagonal and an epistemological dynamic. It was the premature death of children with leukaemia that led Royer to invite Raimbault (46) to his unit. Death was also often decisive in initiating interdisciplinary psychological reflection in the areas of maternity and paediatrics.

In neonatology in the 80s, Soulé (47) at the Institut de Puériculture, explored ‘the death-wish in new-born paediatrics’. In the maternity ward, realisation of the little-known devastating psycho-social effects of mourning for a child triggered a slow but definite change in practises from the 90s onwards (48).

However, the propounding of a psychodynamic clinical reflection on motherhood started in France some 10 years earlier, particularly with the inter-disciplinary work by the psychoanalyst Bydlowski in Clamart, in Papiernik’s team (49, 50). As for the psychoanalyst Druon (51) working in Relier’s neonatal medical unit, she adapted Bick’s method of observation (52) to neonatology.

Finally, the local success of these innovating practises and the dissemination of this work *via* publications and congresses provided strong anchor points in favour of triggering awareness of the need for reflection in France on a coherent form of perinatal clinical psychology.

#### 2.1.4. Identity, boundaries and intersections: interdisciplinary, networked and preventive practise

Perinatal clinical psychology is inseparable from its interdisciplinary nature: adult and child psychiatry, gynaecology-obstetrics, neonatology, paediatrics, general medicine, facilities for the care of infants, and the social services constitute the many branches of a network for which the common goal of coherence is its identity.

As has been well demonstrated in its history, perinatal clinical psychology was born from collaboration among a variety of specialists attending parents and foetuses/infants. It was not elaborated by specialists of the psyche alone (psychiatrists and psychologists), but by a panel of professional participants with distinct training qualifications, as illustrated by the reference list of this manual.

Perinatal anamnesis, in the presence of the mother, father and foetus/baby, apprehends human beings in their wholeness, at the cost of the need to move beyond dogmatic divides among caregivers: psychic/somatic, normal/pathological, gynaecologist-obstetricians/paediatricians, prenatal/post-natal caregivers, etc.

The interdisciplinary approach—often fruitful in its conflicting aspects (53)—is certainly very assertively dynamic in the continuum of perinatal activities. The ‘somatic’ specialists/psychologists-psychiatrists collaboration in teams (including private practise) offer a promise of unity if a common preventive orientation can be materialised. This collaboration in no way means outsourcing from the ‘somatic’ specialists to the psychologists-psychiatrists all that relates to relationships, affects and trauma. However, this preventive axis can prove fruitful if there is common ground, where each one of the participants can shed light, reflecting their qualification, their history and their subjectivity.

This shared investment will generate first and foremost indirect collaboration with psychiatrists-psychologists. Corridor conversations, exchanges in the staff room, liaising in offices, and meetings (of the Balint group type) will be the basis for the daily metabolisation of the shockwaves of the vulnerability observed. By suggesting, but without imposing, a more or less formalised questioning on caregivers’ experiences, this multidisciplinary sharing helps give meaning and combats the defensive operational inertia of the body/mind duality, which has been fed by ambient scientism and biassed training courses. As a result, awareness, which always needs to be re-conquered, of the interactive unity of the patient/carer ‘system’ is assuredly the basic substrate enabling the efficacy of the containing function of professionals. The more discreet are the disorders in presence and the more central to interpersonal relationship for patients, the more the caregivers’ perception of this interaction will be a determining factor.

The direct interventions by psychiatrists-psychologists with families will not replace or compensate for failure of the care initiated. They will follow on from a multidisciplinary reflection that is often worth explaining to parents and babies by caregivers, to prepare for mediation. The psychologists’ direct action will therefore be initiated by harvesting a substantial amount of information from professionals who have been made aware of perinatal psychology and psychopathology in parenthood. In return, direct action by the specialist will find its place, as with their colleagues, in constant reference to the common project in the facility. This mutual collaboration in the field is probably a more dynamic argument in favour of a preventive approach than many petitions on principle.

## 3. The ethics of care and prevention

In the best of cases, medical, psychological and social strategies in perinatal units will therefore follow an open epistemological path, resolutely interdisciplinary, rendered dynamic by research, escaping the insistent medical threat of a ‘ready-made’ protocol and any predictive, alienating, scientistic, preventive logic, and will take root in a broad ethical community debate on the meaning of the transmission of our paradoxical humanity, a simultaneous source of fecundity and tragic conflict.

The subject at hand is sensitive because the public health strategies of a state-based society to pre-empt and provide support for bio-psychic risks in key periods of the life of its members are certainly an indicator of its political maturity. This is particularly true of the perinatal period, where the meaning and content of the measures adopted in favour of preventing suffering inherent in ‘becoming a parent’, ‘being born a human’ and to ‘being a caregiver’ are particularly subtle political issues.

It is therefore not surprising that the preventive discourse in the perinatal field encounters ideological issues and favours Manichean caricature. It is constantly open to manipulation by idealists (‘prevention is going to eradicate all suffering’) and to pessimism (‘the apple does not fall far from the tree’). Between the utopia of total control over nature and the fatalism of an evil repetition across generations, the creative impetus in perinatality and prevention faithfully reflects the paradoxical human blend of Eros and Thanatos.

### 3.1. The birth of early prevention

According to pioneers Soulé et al. (54), prevention work with families ‘is based on three key ideas: the notion of early instatement, the consideration of the rules of infant mental hygiene, and trans-disciplinary^3^ aspects, i.e., the mode of participation of all medical and psychosocial professionals who intervene with families for whatever reason’.

In line with this, prevention is said to be early if it is situated before the child’s birth, at the crossroads between primary prevention (reducing the incidence of an illness) and secondary prevention (decreasing its prevalence).

These basic notions are inseparable from those of risk and vulnerability. Risks relate to ‘the uncertainty of the outcome of a child’s confrontation with environmental or interior stressors’ (55)—and to vulnerability: ‘faced with the same risks, ultimately not all children present the same disorders (…) personal factors play a considerable role and thus lead to greater or lesser vulnerability’.

The notion of vulnerability (56) illustrates the fact that different people faced with a given risk do not present the same disorders. Vulnerability highlights personal factors. The same blow to three dolls made of glass, plastic and steel will not have the same effect. With the concept of resilience popularised today, this concept merits attentive critical analysis (57–59).

However, more than anything, it is the confrontation with the notions of risk indicators and the emergence of hidden demands that could be the most fruitful and plead in favour of a natural, convergent perspective between social, psychological and medical prevention and provision of support for parents or parental substitutes and children. ‘Behind temporary needs’, explained Soulé and Noël, there are situations or behavioural modes among parents or children whose experience shows that the children’s entourage has difficulty in integrating at once the demands of the child, their own needs and social constraints. These are in fact inadequate, conventional or ill-adapted behaviours. This is why they can be ‘warning lights’ (55). The perception of these explicit or implicit warning lights starts with the recognition of the multiplicity of expressions of psychosocial distress.

Finally, as these authors stress, when we envisage risk indicators, ‘warning lights’ and hidden needs, it is important to remember that they are possible signs of distress and in no way obvious symptoms of a recognised pathology. The borderline between well-tempered prevention and an alienating and suspecting investigation is narrow. The challenge for consultants and the whole of the team is to be simultaneously respectful of each person’s freedom and potential, and to be able to hear, not only spoken demands for help, but hidden demands too.

According to Soulé and Noël, the difficulties encountered by the caregivers to reach and maintain this balance is an insistent reminder of how ‘a certain number of motivations supporting us in this fine idea of prevention are also based on the myth of superpower and man’s total control over nature’. If it is not individually and institutionally elaborated, this ideological position ‘becomes harmful if, in the name of health, it introduces rigid regulations for education and behaviour’ (55).

On a larger scale, social control by the state or by a dominant class, the correction of deviance, normalisation, and the totalitarian deprivation of individual freedom are considered by these pioneers as theoretical and excessive arguments against preventive action, but it is primordial not to set them aside, as they have the advantage of pointing the finger ‘at the dangers of doctrines or policies that seek to apply prevention but without assessing the risks and potential excesses’ (55).

### 3.2. Criticism of preventive reasoning: from suspicion to mutually enlightened watchfulness

In a book on perinatal prevention, which showed great maturity in its theoretical, clinical and ethical reflection, Molenat (60) defends a point of view that enables an assessment of the journey undertaken since these pioneering propositions to reach the current state today.

‘Classic risk factors are a first level of vigilance, although carrying their own poison: detecting what is psychopathological without necessarily seeking the patient’s subjectivity, which means that ready-made responses are needed’. Assessment or screening grids, ‘potentially useful in a period of raising awareness, (…) are reused in sometimes unfortunate ways, while a state of mind based on respect for others should prevail’.

According to F. Molénat, if perinatal prevention is not accompanied in the various networks of professionals by a common ethical goal of respecting parental creative subjectivity, it will be synonymous with institutional abuse, thus favouring the deleterious repetitions that it claims to combat. The subjects concerned by this prevention exclusively dedicated to screening for the psychopathological are, in the long run, ‘defendants’ deprived of attention characterised by ‘consideration’ and ‘solicitude’. Experiments and assessments of prevention programmes show that the essential driving force seems to be the identification aid that a professional at ease in his/her role provides for the families he/she cares for. There is also the particular interest caregivers have in their work, which enables them to mobilise their energy in durable manner.

It is the caregivers’ non-elaborated vulnerability, their uneasiness in inter-professional communication and internal power struggles that deprive professionals of a coherent collective containing function that only a network of clinical reflection can allow. If the main question of ‘how can a professional, without knowledge of it, repeat what distressed parents have experienced in their own construction?’ is not addressed, professional collaboration will keep on building towers of Babel.

By radically drawing away from a culture of repair and opening up towards the anticipation of parental competence, early prevention can find its place in the perinatal world. This objective has a *sine qua non* condition: the professionals’ elaborative support, which alone can break off the fatality of repetition of suffering.

Furthermore, it is by being particularly careful with the usual terms of social and medical policies, that prevention will gain its ethical legitimacy: ‘This approach evidently discards the usual medical and social policy terms. It is no longer a question of screening for distress or depression in the post-partum period, but about re-introducing sufficient humanity and rigour into practises so that everyone finds their place’.

These relevant suggestions, enriched through the years by day-to-day clinical work, active research and inter-professional training, are directed towards perinatal mental health^4^, placing ‘the user at the centre of a system to be renovated’.^5^

Finally, the decidedly necessary criticism of preventive reasoning does not justify its outright rejection or the exclusion of its promoters in the name of some potential deviance. On the other hand, it leads us to consider preventive action as inseparable from an on-going, ethical, clinical reflection on caregiving. The encounter of prevention issues (now classic) with, more recently, informed consent for care provision, currently offers innovative and promising food for thought (61).

At the end of a century marked by the triumph of medicalisation, synonymous in France with a decrease in maternal and infantile mortality, with the transfer of birth to a medical environment and to increased social protection, we find ourselves in a period of mutation in familial functioning.

On the one hand, changes in conjugal relationships, the sharp increase in the number of ‘recomposed’ families, changes in familial and filial rights, and on the other hand, a relative decrease in birth rates, a spacing between births, the frequency in late pregnancies, and the abundance of medically assisted procreation procedures are so many facets of the emergent part of the iceberg, amounting to a relative de-institutionalisation of the family, the classic profile of which has been transformed.

This new dynamic is more globally part of an on-going social mutation, where the shift of power from families and religion to medicine triggers a thinning-out of the former community networks and thus affects the symbolic efficacy of the habitual rites of passage attendant on birth and filiation. In this period of metamorphosis of beliefs and social rites, one of the aspects that is likely to be weakened is that of the establishment of parental identity.

To establish a preventive strategy on maternity wards therefore requires first of all to address the issue of what determines the conditions of existence of symbolic reciprocity in secular rites offered by institutions. Is medical follow-up of pregnancies, births and post-partum the psychic organiser of the parenthood process or, on the contrary, the iatrogenic mediator of alienating scientism? Are there rites of passage that enable—individually and collectively—the violence inherent in this transition to be confronted? Are the usual procedures in maternity wards the object of genuinely informed consent, the guarantee of symbolic efficacy (62)?

## 4. Perinatal clinical psychology: promising heuristic potential!

To date, the legitimacy of perinatal clinical psychology has been globally secured in the field in France, even if some worrying geographical and institutional discrepancies persist across the country. Even though increased means have been granted to medical and psychosocial perinatal prevention, it could benefit from more assertive adherence by politicians and the public to ensure durable public funding, given the collective stakes involved.

In the future, the medical and psychosocial strategies in maternity wards will need to pursue an open epistemological journey, resolutely interdisciplinary and rendered dynamic by research, sheltering from the insistent medical threat of a ‘ready-made’ protocol, from a scientistic, predictive, alienating, and preventive logic, and taking root in a broad ethical community debate on the meaning of the transmission of our paradoxical humanity, at once a source of fecundity and of tragic conflict. Quite a challenge!…where clinicians calling on individual or group psychoanalysis have a major role to play in the theoretical and clinical field, remarkable for its heuristic potential.

In fact, in the last 2 decades, innovative clinical experiments in the perinatal period and in early infancy, and the original theories that they have produced, prove extremely fruitful for psychic care in general and psychoanalysis in particular.

As a culmination of this contribution, I would like to focus on the roots of this heuristic force of clinical practise in the area of our origins. Paradoxically, it draws its dynamism from the constancy and ruthlessness of its internal tensions, which call for insistent cross-sectional questioning. Here is an introduction to its main themes:

- Conflict in inter-disciplinary work between ‘somatic’ and ‘psychic’ specialists. It is an inexhaustible fountain of this clinical and epistemological prodigality. The oppositions between the various ‘somatic’ specialists (the frontline between obstetricians and paediatricians and also between ‘somatic specialists’ of the prenatal and postnatal periods) are less often mentioned, but should nevertheless not be underestimated.

Currently, the growing emergence of care and prevention networking strategies is widening the issues of this dynamic towards the intra- and extra-hospital interfaces—public/private, institutional/liberal. The fruitfulness of these confrontations to smooth over the dividing lines is inseparable from the modes of expression and elaboration of the conflicts in presence. In this context, clinical reappraisals, research-action and perinatal training are fertile laboratories favouring an authentic multi-disciplinary culture:

- A permanent dialectic between the discovery of the amplitude of psychological variations and the psycho-pathological forms encountered. In fact, the 1,001 intermingled metamorphoses of becoming a mother, becoming a father and being born a human are a constant recall of the need to put the nosographic categories into perspective, as they are too geometrical.
- An evolving dynamic of the bodily and psychic boundaries of the processes of profound perinatal transformation: they generate elective attention to the issues of limits and the entanglement of the somatic and the psyche, of the self and the other, of the individual and the group, familial, social, etc.
- A reliving of the conflicts of separation punctuating our lives. ‘Psychic transparency’ is too often idealised: it is, at one extreme, a source of morbid and catastrophic repetitions and at the other a source of maturing radical transformations, but in both cases, always synonymous with crisis.
- A re-edition of the *Oedipus complex*, but first and foremost a confrontation with the reminiscence of the classic archaism described by psychoanalysis, and even more, the archaic archaism that I have attempted to describe with my suggestion of the virtual object relationship in intra-uterine life characterised by the issues of the relationship container/content (53).
- A trivial face-to-face with the limits of our symbolic efficacy confronted with the violence of the suspense, the uncertainty of human genesis in real time around embryos/foetuses/babies, and afterwards in the adults around them. This generational threat is by essence potentially traumatic. It concerns any perinatal journey from the most trivial in appearance, in obstetrical and/or psychological terms, to the most blatantly pathological on somatic and/or psychic level.
- Awareness—not without moral violence—of the determination of the many layers conditioning the quality of the societal, institutional and familial containing functions attendant on these metamorphoses of becoming a parent, being born a human and being a caregiver.
- Finally, perinatal clinical practise is confronted with the origins of a human being, in a given culture, both individual and group-based. The subject belongs to a genealogy that pre-exists and is a singular appearance serving the transmission.

The potential creativity of the perinatal world is incarnated in this bitter, often violent dialectic tension between these two poles of origin and originality. This individual specificity, to express itself, will have to impose itself as a branch on the tree of life where the trunk is the filiation, itself rooted in the Russian-doll succession of generations.

The perinatal world is inhabited by this dual origin of human beings: that of the links—whether clear or unclear—with their genetic matrix, their filiation, their collective heritage, and their culture, and that of the uniqueness of the epigenetics of their being, of their potential originality.

In the end, to defend the preventive potentiality of this period is to seek to promote parental and professional originality and at the same time to underline the insistent threat of repetition, sometimes deleterious, always violent, of the origins.

In this setting gathering users and caregivers, there is a strong convergence between the group formalisation of institutional functioning and what (63) called the ‘group psychic apparatus’ which ‘accomplishes a particular psychic task: that of producing and treating the psychic reality of and in the group’. In fact, any situation in perinatal care belongs to this dialectic uniting the sphere of relationships between the members of a group, and the sphere of the relationships of each member in relation to the group. Indeed, the maternity ward as an institution, the unity of time and place and action, unites all the participants present, users and caregivers. This space–time corresponds to the interweaving of affects and representations of and in the user and caregiver group.

The unity present on the scene of clinical care around our origins is the classic scene of tragedy, unitarian in its temporality, its geography and its action. Given the potential of tragedy to ‘inspire terror or pity’ (definition: Petit Robert) it appears that one of our most spontaneous defence mechanisms as members of a maternity ward team or as researchers is that of isolation, which is achieved by centring only on the reactions of the receivers of messages without taking account of the attitudes and experiences of the senders, i.e., ourselves. The challenge in gaining awareness of this protective positioning is essential, since it enables the establishment of the foundations of an interactive, empathetic common territory between the protagonists, all actors in their particular places in a single tragedy, a ‘situation in which a human becomes painfully aware of a destiny or fatality weighing on his or her life, nature and very condition (definition: Petit Robert)’. Whether destiny or fatality, these are indeed the possible issues to address in this collective representation, which will lead either to a predictive oracle condemning a powerless audience to an inexorable morbid fate, or in contrast to a revelation synonymous with narrative and project (destiny), bringing expectation and life.

The maternity institution, a horn of plenty on the throne of cultural propaganda, finally gives birth to as much darkness as light. Between its celestial showroom and the trivial back-of-the-shop of daily drama (infertility, abortion, miscarriage, perinatal accidents, neonate separation from the mother, prematurity, announcement of a disability, institutional violence, psychosocial distress etc.) the contrast can be enormous (14).

At the centre of the interweaving of Eros and Thanatos, perinatal clinical practise spectacularly stages the encounter between our resilience and our creativity and the dizzying heights of our vulnerability. Perinatal clinical psychology meets the challenge of exploring the somatic and psychic reality of this individual and collective scene, supporting its symbolisation by way of relevant approaches. Quite a challenge indeed!

## Author contributions

The author confirms being the sole contributor of this work and has approved it for publication.

## Conflict of interest

The author declares that the research was conducted in the absence of any commercial or financial relationships that could be construed as a potential conflict of interest.

## Publisher’s note

All claims expressed in this article are solely those of the authors and do not necessarily represent those of their affiliated organizations, or those of the publisher, the editors and the reviewers. Any product that may be evaluated in this article, or claim that may be made by its manufacturer, is not guaranteed or endorsed by the publisher.

## Footnotes

^1^The term ‘transparency’ could well suggest the illusion of total transparency. This notion would however neglect the irreducible unconscious of both the woman giving birth and those around her.

^2^http://www.marce-francophone.fr

^3^We prefer the term interdisciplinarity. As with generational transmission, clinically described by Grangeon as trangenerational or intergenerational, the collaboration expressed by ‘inter-’ refers generally to mutual exchanges where the unavoidable risk of conflict will lead to debate that will generate symbols and attunement, while collaboration of the ‘trans-’ type, conversely, will suggest dissymmetrical relationships with little or no elaboration of the antagonisms on its boundaries.

^4^Ministère de l’Emploi et de la Solidarité, Ministère délégué à la Santé, report by Piel and Roelandt: ‘De la psychiatrie vers la santé mentale’. Submitted July 2001.

^5^Ministère de l’Emploi et de la Solidarité, Ministère délégué à la Santé, Plan santé mentale ‘L’usager au centre d’un dispositif à rénover’. Report submitted December 2001.
